# Optical responses of a metal with sub-nm gaps

**DOI:** 10.1038/srep22981

**Published:** 2016-03-11

**Authors:** Sang Jun Park, Tae Yun Kim, Cheol-Hwan Park, Dai-Sik Kim

**Affiliations:** 1 Center for Theoretical Physics and Department of Physics, Seoul National University, Seoul 08826, Korea; 2 Center for Atomic Scale Electromagnetism and Department of Physics, Seoul National University, Seoul 08826, Korea

## Abstract

If the size of a metallic structure is reduced to be comparable to or even smaller than the typical quantum-mechanical lengths such as the Fermi wavelength or Thomas-Fermi wavelength, the electronic structure and optical responses are modulated by quantum effects. Here, we calculate the optical responses of a metal with sub-nm gaps using the eigenstates obtained from an effective-mass quantum theory. According to our simulation, the dielectric responses can be significantly modified by tuning the inter-gap distances. Remarkably, sub-nm gaps occupying a 0.3% volumetric fraction can elongate the penetration depth by an order of magnitude in the terahertz regime. We find that the detailed dependences of electron-photon interaction matrix elements on the involved electronic wavefunctions play an important role in the optical responses. The results draw our attention to these recently fabricated systems.

During the past decade, the field of plasmonics using structures of which one or more dimensions are of nm or sub-nm scale has been actively investigated^1^,^2^,^3^. Recently, only a-few-angstrom-wide gaps between two bulk metals have been realized, using graphene as a spacer^4^,^5^,^6^,^7^. The gap is so narrow that the Fermi wavelength or Thomas-Fermi wavelength is comparable to the width.

The Optical conductivity of a conventional metal is reasonably well described by a Drude theory which accounts for the intra-band electronic transitions:


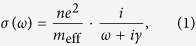


where *e* is the charge of an electron and *m*_eff_ and 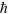
*γ* the effective mass and linewidth of charge carriers, respectively. The key question is how this theory is modified if there are sub-nm gaps in a metal.

In this study, we perform quantum-mechanical calculations, at the level of an effective-mass theory, on the eigenvalues and wavefunctions of electrons in a metal with sub-nm gaps (Fig. 1), from which we evaluate the complex dielectric functions and penetration depths.

The detailed experimental setup such as the thickness along the direction light is propagating [either along *y* or *z* in Fig. 1(a)], the total number of gaps in fabricated structures, etc., are also important in real experiments. Such effects can best be investigated through real-space, finite-difference time-domain simulations. In our study, however, we focus on the fundamental (meta-) material properties rather than on the detailed dependence of the optical responses on the experimental setup. Understanding the properties of a material is the first and the most fundamental step in understanding the optical responses of complex structures made therefrom. Our study provides a physical insight complementary to that which can be obtained from real-space, finite-difference time-domain simulations.

Here, we focus on the optical responses of a metal with sub-nm gaps extended along *y* and *z* [Fig. 1(a)]. We find that, quite surprisingly, even if gaps occupy only a tiny volumetric fraction of a metal, they affect the dielectric responses tremendously. Both the real and imaginary parts of the dielectric function are significantly modified by the gaps. The drastic changes in the optical responses arise from quantum mechanical effects beyond the simple Drude physics.

## Results

### Complex dielectric functions

**Figure 1:**
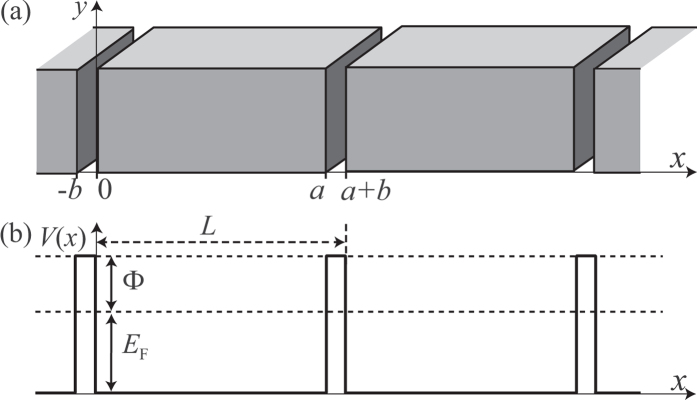
(**a**) The schematic of a metal with gaps extended along *y* and *z* (not shown). (**b**) The schematic of *V*(*x*). Here, Φ is the work function and *E*_F_ the Fermi energy.

**Figure 2:**
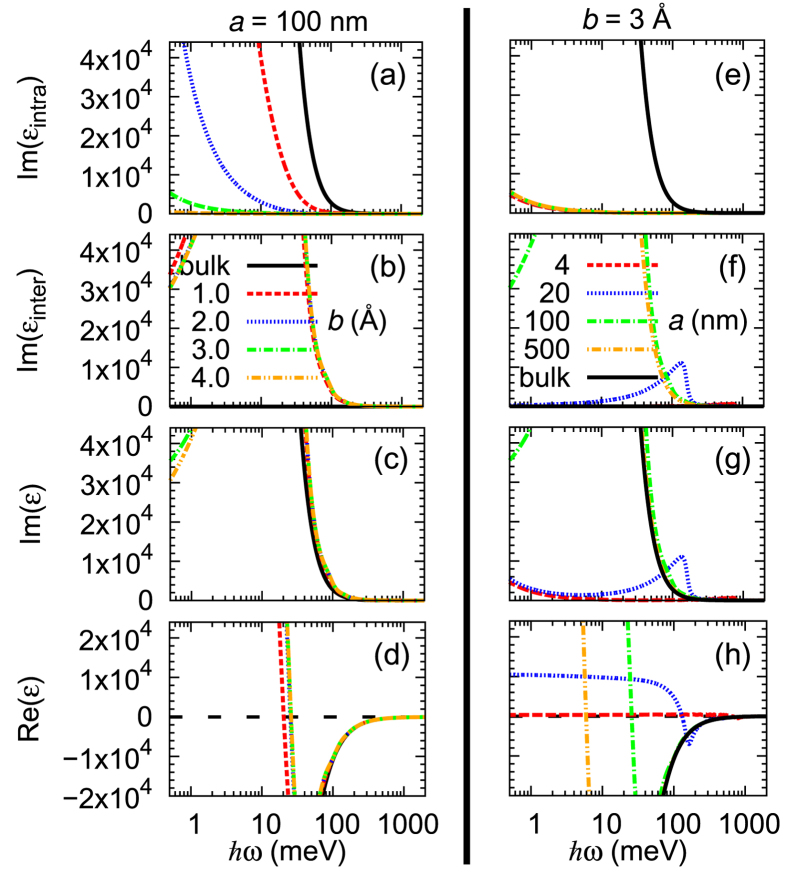
(**a**–**d**) The intra-band contribution [(**a**)] and inter-band contribution [(**b**)] to the imaginary part of the dielectric function and sum of the two [(**c**)] and the real part of the dielectric function [(**d**)] of metals with sub-nm gaps with *a* = 100 nm versus photon energy. (**e**–**h**) Similar quantities as in (**a**–**d**) for sub-nm-gap structures with *b* = 3 Å. In each panel, the solid or black curve represents the results for a bulk metal without gaps.

**Figure 3:**
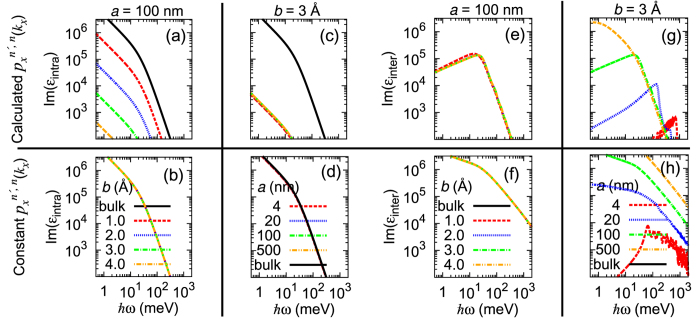
The imaginary part of the dielectric function of metals with sub-nm gaps versus photon energy. Upper panels and lower panels show the results obtained by considering the effects of matrix elements and those obtained by neglecting them, for which we set 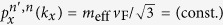 where *v*_F_ is the Fermi velocity. (This choice ensures that the dielectric function of a bulk metal is reproduced).

Figure 2 shows the complex dielectric function (with light polarization along *x*) of metallic structures with sub-nm gaps described in Fig. 1. First, as Fig. 2(a) shows, the intra-band contribution to the imaginary part of the dielectric function, Im*ε*_intra_, decays with *b*, which is exponential as clearly shown in Fig. 3(a). We can understand this exponential decay with *b* by noting that (i) *ε*_intra_ depends only on 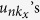 [of a fictitious one-dimensional system characterized by Eq. (6)] with 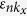 below *E*_F_ [the intra-band sum in Eq. (8) is only over states whose energy is below *E*_F_] and that (ii) *E*_F_ is lower than the potential barrier through which the tunneling amplitude decays exponentially with *b*. (Although a gap of 1 Å is not feasible at the moment, we want to check how the dielectric responses evolve with *b*). As shown in Figs 2(b) and 3(e), the imaginary part of the dielectric function arising from inter-band transitions, Im*ε*_inter_, which is absent in a bulk metal within our model, quickly converges with *b*. Im*ε*(*ω*) of any system with sub-nm gaps shown in Fig. 2(c) is dominated by this inter-band contributions, contrary to the case of a bulk metal. Therefore, importantly, one does *not* need to make a perfect superlattice in order to observe these modulations of optical responses which are dominated by inter-band transitions in a series of isolated slabs. Re*ε*(*ω*) of a metal with sub-nm gaps, which are positive at low *ω*, are also substantially different from that of a bulk metal [Fig. 2(d)].

Since it is clear [Fig. 2(a–d)] that a metal with gaps of 3 Å has essentially the same dielectric properties as a metal with wider gaps, we now consider metals with 3 Å gaps [Fig. 2(e–h)]. Interestingly, Im*ε*_intra_ does not depend on *a* if longer than a few nanometers [Figs 2(e) and 3(c)]. We can understand this insensitivity from Eq. (8) as follows. *ε*_intra_ depends only on the sum (over occupied states) of the diagonal part of the squared optical matrix elements, 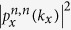, or the squared group velocities. This squared sum is rather insensitive to *a* ≈ *L* 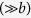 because (i) the size of the Brillouin zone is proportional to 1/*L* and (ii) the gap opening up at the zone boundary or zone center, which is the Fourier component of *V*(*x*) for a proper reciprocal lattice vector *G*_*x*_, is 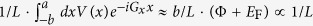.

**Figure 4:**
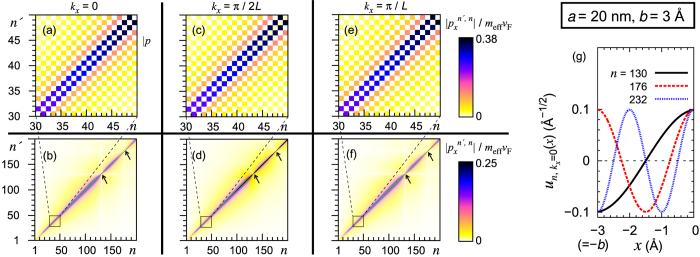
(**a**–**f**) 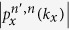 for a metal with sub-nm gaps with *a* = 20 nm and *b* = 3 Å. (**g**) Wavefunctions 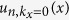 [Eq. (5)] corresponding to the nodes on the diagonal line (*n*, *n*) of 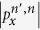 [see the arrows in (**b**,**d**,**f**)].

Figures 2(f) and 3(g) show that for structures with *b* = 3 Å, the peak frequency of Im*ε*_inter_(*ω*) is red-shifted if *a* is increased. In order to understand this behavior, one has to understand the dependence of 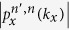 [Eq. (10)] on *n* and *n*′. For simplicity, we assume that *V*(*x*) is much smaller than any other energy scale of the system. If it were not for *V*(*x*), the Bloch state of Eq. (6) at zone boundary *k*_*x*_ = *mπ*/*L* (for an integer *m*) will be just a planewave 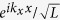. Because of *V*(*x*), the two eigenstates at zone boundary *k*_*x*_ = *mπ*/*L* (for an integer *m*) are of the form 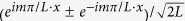. The matrix element involving these two states will be 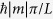 and that involving two states with different *m*’s will be 0. Therefore, 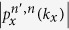 will be non-zero only if 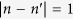. This argument is in qualitative agreement with the results from an actual calculation of the matrix elements [Fig. 4(a,c,e)]; it is clear that the matrix element is biggest if 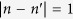. Now, with this simple condition, Im*ε*_inter_ [Eq. (8)] is appreciable when 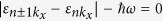 for some *n* from 1 to when 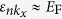. Note that 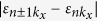 in general increases with *n*. Thus, there exists a maximum frequency in *ω* that satisfies this condition. Also, since the energy spacing between the eigenstates is proportional to 1/*L* ≈ 1/*a*, this maximum frequency in *ω* is red-shifted with *a* [Figs 2(f) and 3(g)].

Figure 2(g,h) show that the dielectric function of a metal with sub-nm gaps is markedly different from that of a bulk metal and also is dependent heavily on *a*.

### Electron-photon interaction matrix elements

We show in Fig. 3 that the quantum-mechanical momentum matrix elements play an important role in the dielectric responses of metals with sub-nm gaps. The dielectric responses obtained under an assumption that the matrix elements 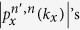 are constant are remarkably different from the results obtained without such an assumption. Especially, the exponential decay in Im*ε*_intra_ with *b* [Fig. 3(a)] is completely absent if matrix elements are assumed constant [Fig. 3(b)]. Also, the shift in the frequency position of the peak appearing in Im*ε*_inter_ [Fig. 3(g)] is not reproduced at all if constant matrix elements are used [Fig. 3(h)] because that shift directly reflects the matrix element 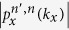 being largest when 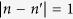 as shown in Fig. 4.

In Fig. 4(b,d,f), we further show that the matrix element 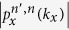-versus-(*n*′, *n*) relation has nodes in the diagonal line *n*′ = *n*. Those nodes (insensitive to *k*_*x*_) at *k*_*x*_ = 0 correspond to the wavefunction 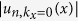 being maximal at the boundaries *x* = −*b* and *x* = 0 and at their periodic replicas [Fig. 4(g)].

## Discussion

**Figure 5:**
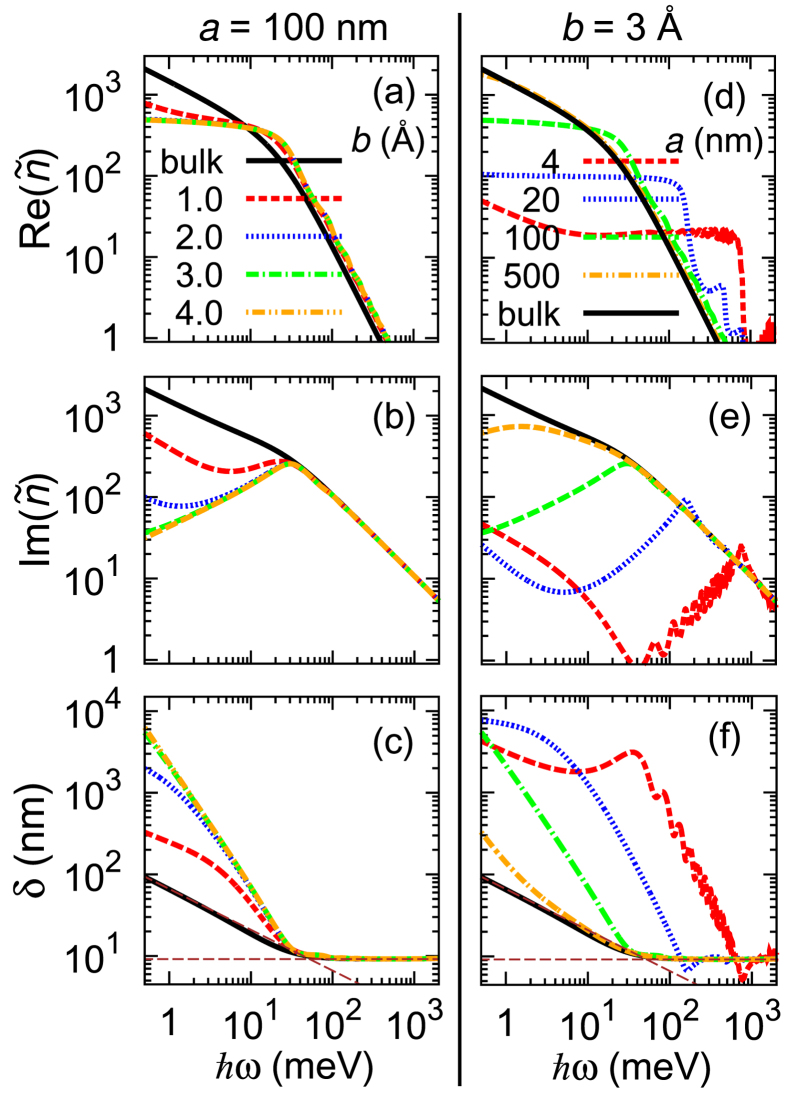
The real and imaginary parts of the refractive index 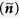 and penetration depth *δ* of metals with sub-nm gaps versus photon energy. The two dashed lines in (**c**,**f**) are the asymptotes in low-frequency regime where 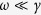 (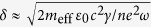: note that the slope in log-log scale is −1/2) and in high-frequency regime where 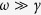 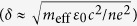.

Using the calculated dielectric function, we obtain the complex refractive indices and penetration depths of metals with sub-nm gaps [Fig. 5], which are remarkably different from those of a bulk metal. Especially, the penetration depth converges with *b* once it is longer than 3 Å and it depends very sensitively on *a* even if *a* is longer than 100 nm; the penetration depth for terahertz waves (with energy 1–10 meV) of a metal with sub-nm gaps (*a* = 100 nm) is about one order of magnitude longer than that of a bulk metal, even if the volumetric fraction of the gaps is only 0.3%.

In conclusion, we reported the optical responses of a metal with sub-nm gaps calculated by using the energy eigenvalues and wavefunctions of electrons from an effective-mass quantum theory. The results are such that the dielectric responses of a metal can be significantly tuned by sub-nm gaps. Considering that metals with sub-nm gaps have already been fabricated and that there is an enormous room for developing the fabrication techniques thanks to the research activities on two-dimensional materials, this great tunability of the dielectric responses predicted in our study draws attention to these new, fascinating systems.

## Methods

In order to account for the effects of sub-nm gaps on the optical responses, we consider metal-gap superlattice structures (the assumption of being periodic along *x* are for computational convenience; we have shown that the results are *not* affected by this assumption) shown in Fig. 1, whose Hamiltonian is


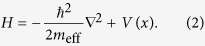


Here, *V*(*x*) is a periodic potential with spatial period *L* = *a* + *b* and, in the range –*b* ≤ *x* < *a*, is given by


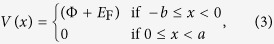


where Φ is the work function and *E*_F_ the Fermi energy. The eigenvalues and eigenstates of this Hamiltonian with band index *n* and Bloch wavevector **k** are given by


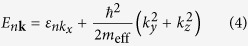


and


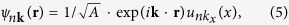


respectively, where *A* is the area in the *yz* plane and 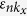 and 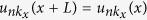 satisfy


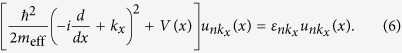


Using the results in Eqs (4) and (5), we calculate the complex, dynamical dielectric function, which, for a non-magnetic system in the case where the polarization of light is along *α* (*α* being *x*, *y*, or *z*), reads^8^,^9^,^10^


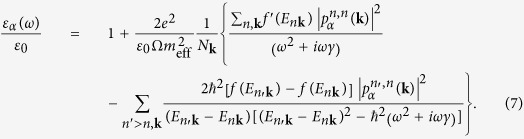


Here, the first term and the second term inside the curly bracket are the intra-band and the inter-band contributions, respectively. *ε*_0_ is the vacuum permittivity, Ω the volume of a unit cell and *N*_**k**_ the total number of **k** points in the Brillouin zone. Also, *f*(*E*) = {exp[(*E* − *E*_F_)/*k*_B_*T*] + 1}^−1^ is the Fermi-Dirac occupation factor at temperature *T*, *k*_B_ the Boltzmann constant and 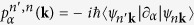 the matrix element of the momentum operator. We use the same linewidth for both the intra-band and inter-band processes. [Note that Eq. (7) does not include the so-called local-field effects, the investigation of which is beyond the scope of this work and is left as a direction for our future research].

After summing over *k*_*y*_ and *k*_*z*_, we find that Eq. (7) for our system reduces to


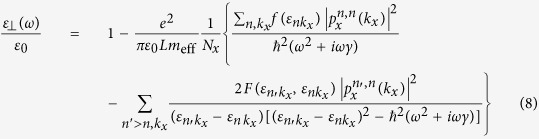


for the polarization of light perpendicular to the gap plane (i. e., along *x*) and


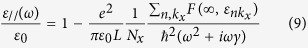


for that within the gap plane (along *y* or *z*). Here, *N*_*x*_ is the number of *k*_*x*_ grid points (*k*_*x*_ = 2*π*/*L* · *n*_*x*_/*N*_*x*_ where 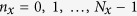),


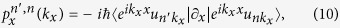


and


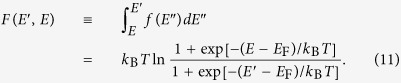


Note that *ε*_//_(*ω*) depends on the eigenvalues only and is almost the same as that for a bulk metal. The matrix elements, 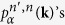 [Eq. (7)] are irrelevant to *ε*_//_(*ω*). We therefore discuss *ε*_⊥_(*ω*) only and drop the subscript _⊥_.

We consider copper (*m*_eff_ ≈ *m*_*e*_ where *m*_*e*_ is the free electron mass) for which the structures hosting sub-nm gaps have been fabricated^7^, we use 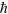
*γ* = 0.026 eV, which reproduces the bulk metallic conductivity at room temperature if plugged in Eq. (1), and We set *E*_F_ = 7.0 eV and Φ = 4.9 eV [see Fig. 1(b)], as in copper. We have checked convergence with respect to *N*_*x*_. Even if we can simulate a finite thermal broadening *k*_B_*T* at no additional cost, we have checked that a reasonable thermal broadening, say, from 0 to the room temperature, hardly changes the presented results and we set *T* → 0.

We are considering the situation where the light is propagating toward a direction contained in the plane of the gap. The intensity of light inside the gaps fabricated in recent experiments (e. g., ref. 7) are stronger than in vacuum and not weak enough to observe the quantum-optical effects in the small-number-of-photons regime. Also, there is no lasing mechanism in the system we studied. For these reasons, we have neglected the quantum-optical or field-quantization effects in our study.

## Additional Information

**How to cite this article**: Park, S. J. *et al.* Optical responses of a metal with sub-nm gaps. *Sci. Rep.* **6**, 22981; doi: 10.1038/srep22981 (2016).
